# Social-Cognitive Determinants of HIV Testing Among Tuberculosis Infected Patients in Kassala State, Sudan

**DOI:** 10.3389/fpubh.2021.521511

**Published:** 2021-04-29

**Authors:** Almutaz M. Idris, Rik Crutzen, Hubertus W. Van den Borne

**Affiliations:** ^1^Department of Health Promotion, Maastricht University/CAPHRI, Maastricht, Netherlands; ^2^College of Applied Medical Sciences, Buraydah Colleges, Buraydah, Saudi Arabia

**Keywords:** beliefs, determinants, social-cognitive, HIV testing, confidence interval-based estimation of relevance, TB patients

## Abstract

**Background:** Use of HIV testing and counselling (HTC) services remains low among TB patients in Sudan. Identifying the social-cognitive (sub) determinants associated with HTC uptake is essential before developing interventions to promote uptake. This study aims to assess the sub-determinants of intention to use and actual behaviour of using HTC services among TB patients in Sudan and to ascertain the most relevant beliefs to inform future interventions.

**Methods:** A cross-sectional study was carried out in five health facilities selected randomly in Kassala State. First, a small elicitation study (*N* = 25) was conducted to inform the Reasoned Action Approach (RAA) based questionnaire. A total of 411 TB patients completed the survey questionnaire. Confidence Interval Based Estimation of Relevance analysis (CIBER) was employed to establish the sub-determinants' relevance.

**Result:** The studied beliefs explained 38–52% of the variance in the intention and 20–35% in the behaviour variance. The beliefs that “Using HTC services increases my fear of being tested positive for HIV” and “Using HTC services increases my fear of losing my partner if I have a positive test result” were negatively associated with intentions and use of HTC services; and both were highly relevant for intervention. The belief “If I use HTC services, I would know my HIV status” was positively associated with intentions and use of HTC services. However, it was less relevant for intervention. Perceived susceptibility to HIV infection was not associated with intention and only weakly associated with use of HTC services. Its relevance was low for intervention.

**Conclusions:** The study showed that the social-cognitive beliefs (sub-determinants) vary in their relationship with the intention and use of HTC services among TB patients; with variable relevance for intervention. Interventions to enhance the use of HTC services should address the most relevant beliefs to maximise the effectiveness of interventions. Further studies are needed to identify other relevant sub-determinants of HTC use behaviour.

## Introduction

Tuberculosis (TB) is a public health problem worldwide, particularly in developing countries, and it is categorised within the top ten leading causes of death (1). An estimated ten million people were infected globally and 1.4 million died due to TB in 2019 (2). HIV-infection increases the TB patients' morbidity and mortality by turning latent TB into an active form, increasing the TB relapse rates and risk of death. Also, co-infected TB patients have higher management costs than other patients (3, 4). Therefore, detecting the HIV status of TB patients can improve the survival rate of TB patients and reducing treatment costs (1, 5–7).

Sudan is part of the World Health Organization (WHO) and the Middle East and North Africa (MENA) countries. The country has a population of 41 million, with around two-thirds of them are rural areas' dwellers (Sudan Federal Ministry of Health, 2018). Among Sudanese, TB is a common health problem (8). Poverty among the Sudanese population remains high, with 46% of the population living below the poverty line (9). Poor living conditions and malnutrition associated with poverty may increase the risk of TB infection (10).

In Sudan in 2019, the estimated TB incidence rate was 67 per 100,000 population, and TB related death rate was about 10 per 100,000 (11). At the time of this study in 2017, rates were higher at 77 per 100,000 incidence and 13 per 100,000 mortality. Sudan alone shouldered about 31% of the new HIV infection cases and 27% of the AIDS-related death in MENA countries. The estimated rate of co-infection was 1.8 per 100,000 (12). From 2009, HIV testing has been offered routinely through Provider Initiated Testing and Counselling (PITC) to all TB patients who seek care in Tuberculosis Management Units (TBMUs) with patients having the right to decline testing for HIV (13). The Sudan National Tuberculosis policy stated that every TB patient should be offered HIV testing (Sudan National Tuberculosis Policy, 2013).

The HIV infection rate appears to be high among TB patients in Sudan. A study by Abdallah et al. (14) in Kassala State in Sudan showed that the prevalence of HIV infection among TB patients was 18.3%. Despite this high rate, HIV testing among TB patients in Sudan remains consistently low (15). Increasing uptake of HTC services among TB patients is needed to detect co-infected cases earlier (14).

Previous studies showed that different social-cognitive factors could influence human health behaviours, including HIV testing behaviour. These social-cognitive determinants include attitude, social pressure, perceived facilitators and barriers, and perceived risk of HIV infection (16–18). Without understanding the social-cognitive (sub) determinants, it would be hard to change people behaviours (19–22).

Social cognitive theories, such as the Reasoned Action Approach (RAA), are useful in explaining human health behaviours (21). According to the RAA, behavioural intention is the most proximal predictor of behaviour, and this intention is a product of attitude, subjective norms, and perceived behavioural control. The latter three constructs originated from behavioural beliefs, normative beliefs, and control beliefs, respectively (23). A previous meta-analysis found that attitude, subjective norms and perceived behavioural control accounted for 39% of the behaviour variance and 27% variance in the intention. A previous study investigating the predictors of intention to Voluntary Counselling and Testing reported that attitude, subjective norms, and perceived behavioural control explained 30.3% of the intention's variance (24). The RAA can include other variables such as past behaviour and perceived risk to improve the model's prediction utility. For example, a previous study showed that the inclusion of the perceived risk of HIV infection could increase the prediction of behavioural intention for HIV testing (25).

There is a lack of insight into social-cognitive (sub) determinants of HTC services use among TB patients in Sudan. Therefore, this study employed an RAA to investigate the association between social-cognitive beliefs (sub-determinants), intentions and behaviour to assess their relevance for interventions to enhance HTC services use among TB patients in Kassala State, Sudan.

## Methods

### The Study Design and Settings

This cross-sectional study was carried out in Kassala State. Administratively Kassala is divided into eleven localities, with an estimated population of 2.9 million. In total, there are 22 TBMUs serving TB infected patients in the state. Data from the State TB program showed that about 8,730 TB patients attended these TBMUs in 2017. These sites open in the daytime from 8 am to 3 pm with 2 days specified for referred clinics each week. HIV testing is provided as part of the TB patient standard care in all TBMUs. The HIV testing in the TBMUs is provided under joint and direct administration of the National TB program and the AIDS Control Program. Data were collected from five randomly selected TBMUs in the state; namely Kassala, Rural Kassala, Halfa, Khashim Algirba, and Wed Elhelew TBMUs. The recruitment period was from July 2017 to February 2018.

Well-trained data collectors used a structured questionnaire to collect data from the study participants. Before the interview, data collectors explained the purpose of the study to all participants and then obtained informed consent from them. Children and those who were very ill or did not provide informed consent were excluded from the study. During the interview process, the immediate next one replaced the participant who refused to participate. Ethical approval was gained from the Research Ethical Committee in the Ministry of Health in Kassala State. Permission was received from the State National Tuberculosis Program and the administrative authorities for the selected TBMUs.

### Participants

The eligible participants were all TB patients aged 18 years and over with confirmed TB infection diagnosis who attended the selected five TBMUs in Kassala State during the study period. A three-step random sampling design was used. First, five localities were selected through a simple random sampling method from the Kassala State's total eleven localities. Second, from each locality, one TBMU was chosen randomly. Finally, in each TBMU, on-site systematic random sampling was employed to select the participants.

### Variables and Measurements

In the analysis, the outcome variables were the behavioural intention to use and actual HTC services use. The predictors were behavioural beliefs, normative beliefs, control beliefs, perceived risk beliefs, and past HTC services use behaviour. The behaviour in question refers to using HTC services in the TBMU in the next 3 months, and learning their HIV status.

A written questionnaire was used to collect socio-demographic and social-cognitive variables related information from the participants. The socio-demographic data included age, gender, residence, education level, working, and marital status.

The social-cognitive variables were assessed to be congruent with HTC use behaviour in TBMUs in the next 3 months. The social-cognitive variables questions were developed based on similar previous studies (26) results and findings from a beliefs elicitation study. The elicitation study was conducted among a small group (*N* = 25) of the study population to identify their behavioural beliefs, normative beliefs, and control beliefs regarding HTC services use. The most commonly mentioned salient beliefs were included in the final survey questionnaire.

The intention to use HTC services was assessed by asking the participants to indicate how likely they intend, want, and expect to use HTC services in the next 3 months. Answers ranged from unlikely (+1) to likely (+7).

The actual use of HTC services was assessed after 3 months by asking the participants who had completed the questionnaires to indicate whether they did use the HTC services or not. The participants' responses were verified through their medical records in the TBMUs. The medical record information was taken if there was a discrepancy between the patient's response and their record.

The behavioural beliefs were examined by asking the participants to indicate to which extent their use of HTC services in the next 3 months is likely to be influenced by the following statements: they would know their HIV status, protect themselves from getting HIV infection in the future, increasing their fear of being tested positive for HIV, prevent transmitting HIV infection to their family, and increasing their fear of losing their partner if they tested positive. Responses ranged from unlikely (+1) to likely (+7).

For normative beliefs, respondents were asked to rate the extent to which they believed that their friends, counsellor, partner, and doctor think that they should use HTC services in the next 3 months. Responses were rated on a 7-point bipolar agree-disagree scale.

The control beliefs about HTC services use were assessed by asking the participants to rate five salient beliefs: “I have enough money to reach HTC services,” “If I use HTC service, my health care providers will keep the test result confidential,” “If I think about using HTC services, I feel scared about disclosing the positive test result,” “If I use HTC services and tested positive for HIV, I could have treatment,” and “Using HTC services increases my fear that people would assume I am infected with HIV.” Participants responses ranged from unlikely (+1) to likely (+7).

Two items were used to measure the perceived susceptibility to HIV infection. The participants were asked to indicate how likely they believe themselves or their partners may be infected with HIV infection, and how likely their friends may be infected with HIV. Answers were arranged on a 7-point scale. The past behaviour was measured by asking the participant whether they had attended HTC services during the last year. Responses ranged from disagree (+1) to agree (+7).

### Study Size

A pre-specified confidence interval for correlation values (27) was used to estimate the sample size. By taking 0.05 as a correlation coefficient and confidence interval half-widths at 0.10, the required sample size was 383. Then 15% was added to cater for refusal. Therefore, the final sample was 441 participants.

### Statistical Analysis

The Statistical Package for Social Science (SPSS) version 21 and R version 3.6.1 were used for data analysis. Descriptive characteristics included the distribution of the socio-demographic variables.

A Confidence Interval Based Estimation of Relevance (CIBER) (28) was used to establish the relevance of sub-determinants (social cognitive beliefs in this study) for interventions targeting intention and use of HTC services. CIBER is a data visualisation method whose output consists of two panels with diamond shapes.

In the left-hand panel, the diamond shows the sub-determinant's mean with a 99% confidence interval. The diamond fill colour gives information about the item's mean: the redder indicates a lower mean, and the greener colour indicates a higher mean. The blue colour indicates the item with mid-scale mean.

The right-hand panel shows diamonds indicative of the strength of association (correlation coefficients) between the sub-determinants and dependent variables (intention and use of HTC services in this study) with 95% confidence intervals. The right-hand diamond fill colour is indicative of the nature of the association: redder diamond indicates a strong negative association, greyer diamond indicates a weaker association, and greener diamond shows a strong positive association. At the top of the plot, CIBER provides the explained variance in outcome variables by all sub-determinants.

Data visualisation has three advantages in the context of determinant selection. First, visualisation enables mapping the data onto spatial dimensions, facilitating comparisons, which are necessary when making selections. Second, visualisation foregoes the seeming accuracy and objectivity afforded by numbers (29). Given the relative width of most sampling distributions and the subsequent variation that occurs in estimates over samples (30), caution in basing decisions on the exact computed numbers seems prudent. Third, visualisation enables assessing confidence intervals for means in the context of the raw data. In short, CIBER acknowledges that several metrics need to be combined and interpreted in order for data to become valuable information for selecting determinants.

## Results

The total number of the participants was 441 TB patients attending five selected TBMUs in Kassala State. The refusal rate among the eligible participants was 3.5%, and the main reason mentioned was lack of time.

Table 1 shows the socio-demographic variables of the study participants. Of the total participants, 29.2% were in the age group 20–29 years, and 23.8% in the age group 30–39 years. Males accounted for 58.7% of the participants. The majority of the respondents (63.5%) were from rural areas. Among the participants, 43.3 % were illiterate, 19.5% received informal education, and 37.2% had primary school or above. Almost seventy per cent of the participants were ever married, and approximately half were not working. About fifteen percent (14.7%) of the participants reported using HTC services in the following 3 months and learned their HIV status.

**Table 1:** Socio-demographic characteristics of study participants (*N* = 441).

**Variable**	***n***	**%**
**Gender**		
Female	182	41.3
Male	259	58.7
**Age group**		
Below 20	14	3.2
20–29	131	29.7
30–39	105	23.8
40–49	88	19.9
50–59	77	17.5
60 and above	26	5.9
**Residence**		
Urban	161	36.5
Rural	280	63.5
**Education level**		
Illiterate	191	43.3
Informal education	86	19.5
Primary school	76	17.2
Higher secondary school and above	88	20.0
**Working status**		
Not working	217	49.2
Working	224	50.8
**Marital status**		
Never married	136	30.8
Ever married	305	69.2
**Testing status**		
Tested	65	14.7
Not tested	376	85.3

**Figure 1:**
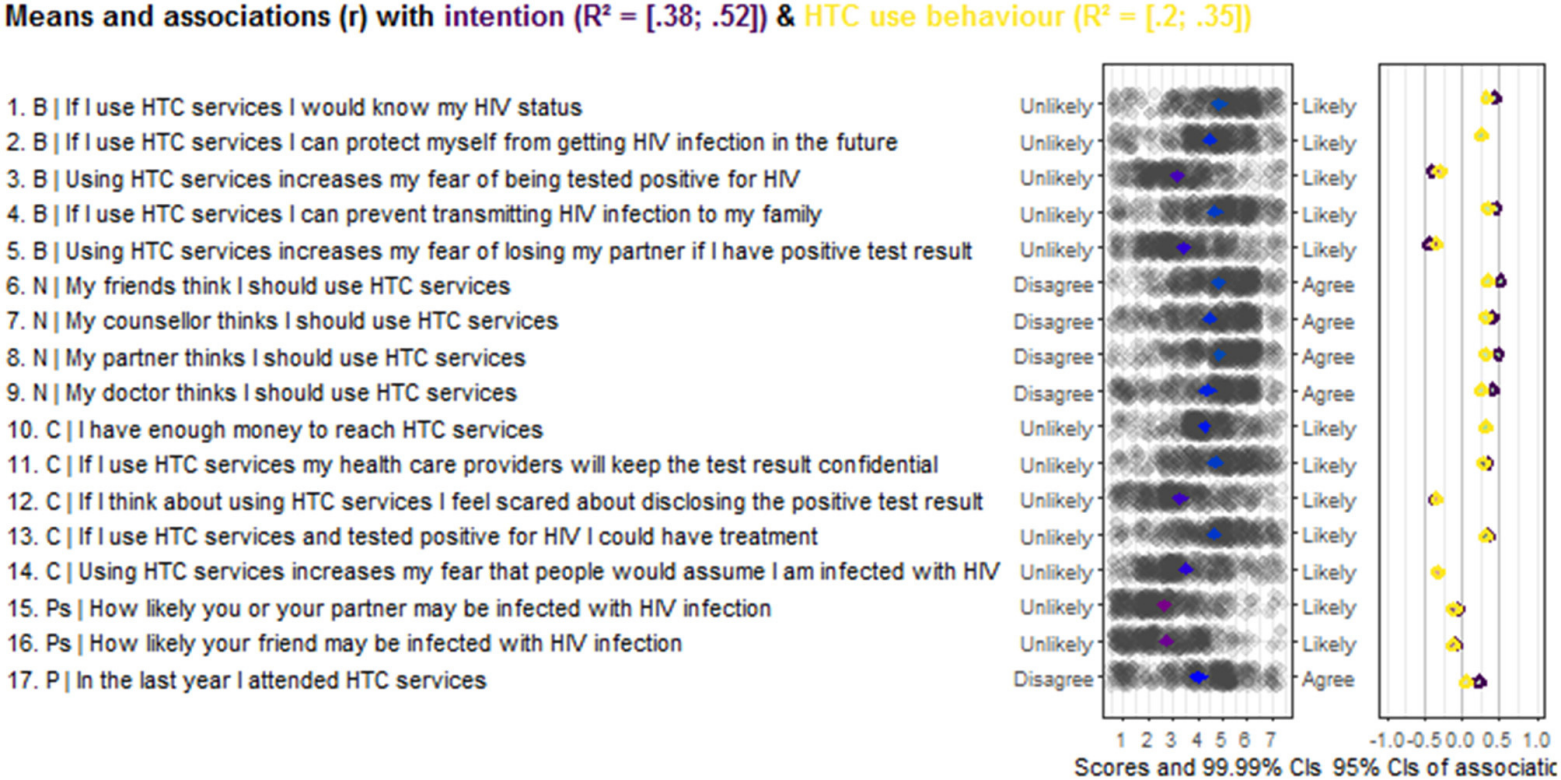
Output CIBER regarding Social-Cognitive beliefs of the intention and use of HTC services behaviour among TB in Kassala State, Sudan (*N* = 441). Behavioural beliefs; N, Normative beliefs; C, Control beliefs; Ps, Perceived Susceptibility; P, Past behaviour.

Figure 1 presents the CIBER analysis results. The studied social-cognitive beliefs explained 38% to 52% of the intention's variance and accounted for 20 to 35% of the HTC use behaviour variance.

The results indicated that the belief “If I use HTC services, I would know my HIV status” has a positive association with intention and HTC use behaviour and its scores are in the upper part of the panel. The belief “Using HTC services increases my fear of being tested positive for HIV” has scores in the lower panel showing a strong negative relationship with intention and behaviour. The participant's belief that “If I use HTC services, I can prevent transmitting HIV infection to my family” has a strong positive relationship with the intention and the behaviour, and its item scores are relatively high. The mean score of the belief “Using HTC service increases my fear of losing my partner if I have positive test result” is located in the middle of the scale and negatively associated with both intention and behaviour regarding HTC services use.

The respondents' beliefs that their friends think they should use HTC services is positively associated with intention and behaviour, with scores on the upper end of the scale. The participants who believed that their counsellor or their doctor thinks they should use HTC services showed a positive association with intention and behaviour, and the scores are relatively above the middle of the scale. The mean score of belief “My partner thinks I should use HTC services” is relatively high, and it has a positive association with the intention and HTC services use.

Also, the CIBER results show that the belief about confidentiality of the test result is positively associated with the intention and HTC service use behaviour. It scored in the upper half of the panel. The belief “If I use HTC services and tested positive for HIV, I could have treatment” scores in the upper part of the scale and is positively associated with intention and HTC services use. The scores for the items “If I think about using HTC services, I feel scared about disclosing the positive test result” and “Using HTC services increases my fear that people would assume I am infected with HIV” are in the middle of the scale, and both are negatively associated with intention and behaviour regarding HTC services use.

Our results also indicate the belief “How likely you or your partner may be infected with HIV infection” is not associated with intention and weakly associated with HTC behaviour, scoring below the middle of the scale. The item “How likely your friend may be infected with HIV infection” is also not associated with intention and use of HTC services. Participants who in the last year had attended HTC services were positively associated with intention, but not with behaviour to use HTC again.

## Discussion

This study aimed to investigate the association between social-cognitive beliefs and the intention and use of HTC services by TB patients, and to assess the relevance of these beliefs for developing future interventions to enhance HTC services use behaviour. Our study results showed that the proportion of participants who use HTC services and tested for HIV infection is relatively low (14.7%), which is in line with the national HIV testing rates among TB patients in Sudan. The study suggests that the social-cognitive beliefs have variable relevance for interventions to enhance intentions and use of HTC services among TB patients.

Based on all the social cognitive beliefs included in the study, the explained variance in the intention and HTC use behaviour ranged from 38 to 52% and 20 to 35%, respectively. This result fits with Armitage and Conner's meta-analysis (31), in which they reported an explained variance of 27% in the behaviour and 39% in the intention.

A study from Uganda (32) reported an association between Voluntary Counselling and Testing use and the need to know HIV status. Our study results suggested that the belief “If I use HTC services, I would know my HIV status” was strongly and positively associated with the intention and use HTC services with scores in the upper scale. A high mean score indicates that participants are already convinced that by using HTC services, they become aware of their HIV status. This combination makes it less relevant for targeting this belief with an intervention.

Our study results also suggest that the participants' belief “If I use HTC services, I can prevent transmitting HIV infection to my family” had a strong association with the intention and behaviour of HTC services use. This agreed with a study among college students in the United States (33). However, participants' scores are in the upper part of the scale which suggests that they believed that using HTC services results in preventing their family from getting HIV infection. This combination leaves little room for improvement, making it a less relevant belief to be selected. Nevertheless, this belief can still be reinforced and can be aimed at participants who are not convinced that the use of HTC services prevents the spread of HIV infection.

In this study, the belief “Using HTC services increases my fear of being tested positive for HIV” showed a strong negative relationship with the intention and actual use of HTC services and its scores are roughly distributed in the middle of the scale. This combination makes it a relatively highly relevant belief, suggesting that it needs to be targeted by an intervention. This might suggest messages that focus on the benefits to individuals if they learn their HIV status, and in this way we can reduce their fear of receiving a positive test result.

The belief “Using HTC services increases my fear of losing my partner if I have a positive test result” showed a strong negative relationship with the intention and use of HTC services, scoring middle of the scale. This pattern makes it a viable candidate to be targeted by interventions. HIV related stigma is well-documented as a barrier to accepting and using HTC services in different settings (34–36). A previous study (37) conducted among TB patients found that fear of HIV related stigma was a reason for low use of HIV testing, and our finding shows that fears of a positive HIV result and fears of losing their partner if tested positive are both relevant for intervention among TB patients. This implies that HTC services use can be improved by tackling these fears.

This study found a strong positive association of the belief “My doctor thinks I should use HTC services” with the intention and HTC services use. This finding suggests that doctors can influence the TB patients' acceptance and uptake of HTC services. The role of doctors in HIV testing decisions was suggested in a previous study in Sudan among pregnant women (18). However, this belief's relevance in terms of intervention is relatively low, as the scores were in the middle of the scale indicating that nearly half the participants were persuaded that their doctors believe that they should use the HTC services. It could still be possible to target interventions to convince the other half to change their perception.

The belief “My friends think I should use HTC services” has a significant positive association with the intention and behaviour of using HTC services. A previous study conducted among adolescents showed that peer pressure was associated with HIV testing behaviour (38). The belief with a mid-scale mean indicates that many participants believe that their friends think they should use HTC services. However, the belief can be considered to have relatively low relevance for intervention.

Our results also suggest that the belief “If I use HIV Testing and counselling service, my health care providers will keep the test result confidential” was positively and strongly associated with intention and behaviour regarding HTC services use. This finding is supported by previous studies conducted in Uganda (39) and Northern Tanzania (40), which reported confidentiality as an essential factor that can affect the HIV testing service uptake. The relatively high mean scores of the beliefs reflects that many participants believe that health care providers will maintain their test result confidential.

Our study shows that the score for the belief “How likely is it that you or your partner may be infected with HIV infection” is in the lower part of the scale which implies that most of the participants do not perceive themselves and their partner to be susceptible to HIV infection. This result disagrees with a study conducted in Sudan (18) which reported high perceived susceptibility among its participants. Results of previous studies on the relationship between perceived susceptibility of HIV infection and HIV testing are controversial. Some studies showed association (25), while others suggested no association (24, 41). In our study, this belief is not associated with the intention to use and weakly with HTC use behaviour.

There are some limitations to our study, and they will need to be addressed in further studies. The use of self-reported data in the study can be affected by social desirability bias. Previous exposure of some of the study participants to the HTC services interventions may have resulted in the positive effect of some beliefs regarding the use of the HTC services. We believe that social desirability bias has been minimised by referring to the patients' medical records.

The questionnaire did not include all other possible beliefs related to HTC services use, but it focused, as recommended by the RAA methodology, on the most salient beliefs that the participants reported in the elicitation study. An elicitation study provides important information on what individual beliefs are prominent regarding the behaviour of interest.

In conclusion, our study suggests that different beliefs among TB patients influence their intention and use of HTC service. Fear of testing positive for HIV and losing their partner if tested positive are strongly negatively related to intentions and behaviours to use HTC services, and they are highly relevant to be selected for targeting in future interventions. The belief “If I use HTC services, I would know my HIV status” was positively associated with intention and behaviour, but it has a low relevance because there is little room for improvement. The same goes for the belief, “If I use HTC services, I can prevent transmitting HIV infection to my family.” Perceived susceptibility to HIV infection was not associated with intentions and only weakly associated with HTC use behaviour. The relevance of participants beliefs about their doctors, friends, and confidentiality of the test results regarding HTC services use was relatively low.

The study was carried out in a particular geographical area in Sudan and this may lead to a possible bias in the generalisability of the study results to the TB population across the country. Since our participants are demographically quite similar to other TB patients in Sudan, they may not differ much in what they believe about HIV infection and HTC services. We believe that our study results can be used for other TB patients in Sudan and these insights can be relevant to other settings in Sudan. National TB programme interventions that target enhancing HTC services use should address the highly relevant beliefs first to have better results for influencing positive behaviour change.

## Data Availability Statement

The datasets generated for this study are available on request to the corresponding author.

## Author Contributions

AI, RC, and HWB developed the study concept and design. AI collected the data. All authors analyzed the data, interpreted the results, drafted the manuscript and discussed and agreed on the final version of the manuscript.

## Conflict of Interest

The authors declare that the research was conducted in the absence of any commercial or financial relationships that could be construed as a potential conflict of interest.
